# The changing clinical profile of celiac disease: a 15-year experience (1998-2012) in an Italian referral center

**DOI:** 10.1186/s12876-014-0194-x

**Published:** 2014-11-18

**Authors:** Umberto Volta, Giacomo Caio, Vincenzo Stanghellini, Roberto De Giorgio

**Affiliations:** Department of Medical and Surgical Sciences, University of Bologna, S.Orsola-Malpighi Hospital, Bldg #5 Via Massarenti 9, 40138 Bologna, Italy

**Keywords:** Celiac disease, Natural history, Clinical features

## Abstract

**Background:**

Celiac disease is a multiform, challenging condition characterized by extremely variable features. Our goal was to define clinical, serological and histopathological findings in a large cohort of celiacs diagnosed in a single referral center.

**Methods:**

From January 1998 to December 2012, 770 patients (599 females, median age 36 years, range 18-78 years) were diagnosed as celiacs at St.Orsola-Malpighi Hospital (Bologna, Italy). The clinical phenotypes were classified as: 1) classical (malabsorption syndrome); 2) non-classical (extraintestinal and/or gastrointestinal symptoms other than diarrhea); 3) subclinical. Serology, duodenal histology, comorbidities, response to gluten-free diet and complications were evaluated.

**Results:**

Disease onset was symptomatic in 610 patients (79%), while 160 celiacs showed a subclinical phenotype. In the symptomatic group the non-classical prevailed over the classical phenotype (66% vs 34%). Diarrhea was found in 27%, while other gastrointestinal manifestations were bloating (20%), aphthous stomatitis (18%), alternating bowel habit (15%), constipation (13%) and gastroesophageal reflux disease (12%). Extraintestinal manifestations included osteopenia/osteoporosis (52%), anemia (34%), cryptogenic hypertransaminasemia (29%) and recurrent miscarriages (12%). Positivity for IgA tissue transglutaminase antibodies was detected in 97%. Villous atrophy was found in 87%, while 13% had minor lesions consistent with potential celiac disease. A large proportion of patients showed autoimmune disorders, i.e. autoimmune thyroiditis (26.3%), dermatitis herpetiformis (4%) and diabetes mellitus type 1 (3%). Complicated celiac disease was very rare.

**Conclusions:**

Our study demonstrates that the clinical profile of celiac disease changed over time with an increasing rate of non-classical and subclinical phenotypes.

## Background

Until the end of the second millennium, the classic view of celiac disease (CD) was that of a rare food intolerance characterized by villous atrophy and overt malabsorption mainly affecting pediatric patients. Recently, CD has markedly changed due to considerable advances in the knowledge of its pathogenic and diagnostic aspects [1,2]. CD is now an established autoimmune disorder triggered by gluten which activates an immune reaction against the CD autoantigen, i.e. tissue transglutaminase (TG2), in genetically predisposed subjects [3]. The genetic susceptibility to CD is confirmed by its occurrence in about 10% of first-degree relatives and by its close linkage with histocompatibility leukocyte antigens (HLA)-DQ2 and -DQ8 [4]. Environmental factors such as breastfeeding, timing of weaning, viral/bacterial infections and microbiota changes can play a role in the onset of CD at any age [5-8].

The identification of biomarkers, e.g. antibodies to endomysium (EmA) [9] and to TG2 (anti-TG2) [10], has changed the epidemiology of CD from a rare to a frequent condition with an expected prevalence higher than 1% in the worldwide population. Nonetheless, the majority of patients with CD remain undiagnosed leaving the celiac ‘iceberg’ still submerged [11]. Serological screening has allowed an early CD diagnosis in its preclinical stage with the result that symptom presentation has radically changed compared to the past [12,13]. Indeed, CD is less commonly detected in patients with diarrhea, rather it occurs frequently in patients with other gastrointestinal symptoms, i.e. constipation and bloating, as well as with extra-intestinal manifestations and even in asymptomatic patients [14]. The different mode of presentation has led experts to elaborate the Oslo classification which subdivides CD in symptomatic, i.e. “classical” and “non-classical”, vs. clinically silent, i.e. “subclinical”, phenotypes [15].

In this study we retrospectively examined the clinical presentation of a large cohort of consecutive CD adult patients diagnosed in a single Italian referral center during a 15-year period. Our primary goal was to verify whether non-classical and subclinical CD increased over time compared to the classical CD. Furthermore, we aimed to define serology, histopathology, response to gluten free diet (GFD) and occurrence of complications in CD.

## Methods

This is a retrospective paper assessing patients from January 1998 to December 2012: 770 CD patients (599 females, F/M ratio 3.5:1, median age at diagnosis 36 years, range 18-78 years) were consecutively diagnosed at the referral center of St. Orsola-Malpighi University Hospital (Bologna, Italy). All patients included in the study gave written informed consent to publish their own data when they were referred to our outpatient clinic for the first time. The diagnosis relied on duodenal biopsy and serology as well as HLA typing when indicated. Small intestinal biopsies (n = 5 samples), taken from the bulb and the second duodenal portion, were classified according to Marsh-Oberhüber [16]. Serology included IgA anti-TG2 and EmA together with IgG anti-TG2 or deamidated gliadin peptide antibodies (DGP) in the case of IgA deficiency [17]. Genetic testing, performed when there was a discrepancy between histology and serology as well as in selected cases, assessed HLA-DQ2 and-DQ8. The finding of villous atrophy (including partial - 3a -, subtotal - 3b -, total atrophy - 3c) and positive serology confirmed CD, whereas cases with positive serology and normal or mild intestinal lesions (increased intra-epithelial lymphocytes - IEL - i.e. lesion type 1) were classified as potential CD if HLA-DQ2 and/or -DQ8 tested positive. Cases with villous atrophy, negative serology and positivity for HLA-DQ2 and/or -DQ8 were confirmed as CD when a second intestinal biopsy showed villous regrowth after 1-year of GFD.

According to the Oslo classification, patients were classified as: 1) classical CD with malabsorptive syndrome, i.e. diarrhea and weight loss irrespective of extraintestinal manifestations; 2) non-classical CD with gastrointestinal symptoms (except for diarrhea) and extraintestinal manifestations; 3) subclinical CD for clinically silent cases or with symptoms below the threshold of detection [15].

Moreover, CD patients were investigated for pathologies known to be frequently associated with CD (including Hashimoto’s thyroiditis, type 1 diabetes mellitus -DM), autoimmune liver disorders, connective tissue diseases, neurological, allergic and chromosomal disorders) and were followed up for the response to GFD and the occurrence of complications [18].

Since patients were not individually identified, a simplified International Review Board approval by the Ethics Committee of the St. Orsola Malpighi Hospital was obtained.

### Statistical analysis

Statistical analysis was performed by applying Mann Whitney U test to compare: 1) age of patients at diagnosis in symptomatic (classical and non-classical) vs. subclinical CD, as well as classical vs. non-classical phenotypes; 2) age of patients at diagnosis in seropositive and seronegative CD; 3) age of patients at diagnosis in complicated vs. non-complicated CD. Moreover, the Pearson Chi-square test was used to compare the clinical phenotype in seropositive vs. seronegative CD patients and in complicated vs. non-complicated CD. The above-mentioned statistical evaluations were carried out by means of Graphpad Instant Version3.0a (Graphpad Software Inc., San Diego, CA, USA).

## Results

### Clinical data

**Figure 1:**
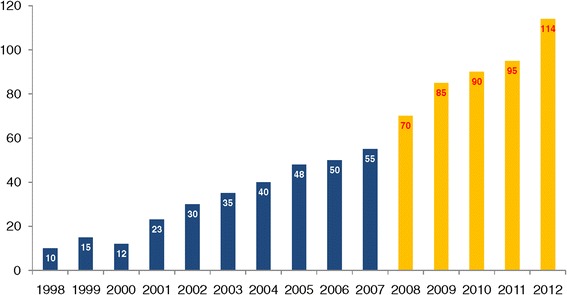
**The annual distribution of CD diagnoses in the referral center of the St. Orsola-Malpighi University Hospital from 1998 to 2012.** Of the 770 diagnosed patients, 318 (41.2%) were identified in the first 10 years (1998-2007), whereas the other 452 (58.8%) were diagnosed in the last five years (2008-2012).

**Figure 2:**
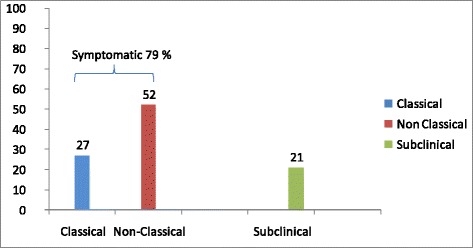
**Prevalence of symptomatic and subclinical phenotypes in the 770 CD patients.** Note that 610 patients (79%) were diagnosed as symptomatic, whereas the remaining 160 (21%) were classified as subclinical CD. Of the 610 symptomatic patients, 210 (34%) displayed the classical onset with diarrhea and malabsorption (regardless of extraintestinal manifestations), whereas the other 400 showed the non-classical form with gastrointestinal symptoms (other than diarrhea) and extraintestinal manifestations.

**Figure 3:**
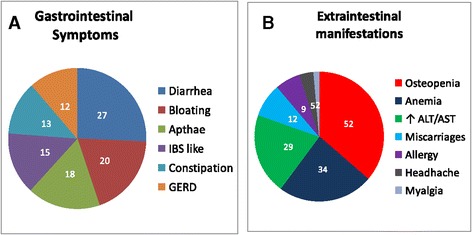
**Gastrointestinal and extraintestinal symptoms in 770 CD patients. A)** Symptoms related to gastrointestinal tract included diarrhea (27%), bloating (20%), aphthous stomatitis (18%), alternate bowel habit (15%), constipation (13%) and gastroesophageal reflux disease (GERD) (12%). **B)** Extraintestinal manifestations, alone or in combination with gastrointestinal symptoms/signs, included osteopenia/osteoporosis (52%), anemia (34%), cryptogenic hypertransaminasemia (29%), recurrent miscarriages (12%), IgE-mediated allergy (9%), headache (5%) and fibromyalgia-like symptoms (2.2%).

The annual distribution of CD diagnoses is shown in Figure 1. Of the 770 CD patients, 318 were diagnosed in the first 10 years (1998-2007), whereas 452 were detected in the last five years (2008-2012). The onset of CD was symptomatic in 610 patients (79%), whereas the remaining 160 (21%) showed a subclinical phenotype. Of the 610 symptomatic patients, 210 had the classical phenotype, whereas 400 displayed the non-classical phenotype (Figure 2). In the period 1998-2007 the classical, non-classical and subclinical phenotype were respectively found in 47.2%, 43.1% and 9.7% of CD cases, whereas in the period 2008-2012, the most frequent clinical phenotype was the non-classical (58.2%), followed by the subclinical (28.5%) and by the classical (13.3%). The median age at diagnosis was significantly lower in patients with the subclinical (32 years) than with the symptomatic phenotype (37 years) (*P* < 0.001). No significant difference was found in the median age at diagnosis between classical and non-classical forms (39 vs. 36 years, *P* = 0.058). Taking into account all CD patients (n = 770), about half (53%) of them had gastrointestinal symptoms/manifestations, i.e. diarrhea (27%), bloating (20%), aphthous stomatitis (18%), alternating bowel habit (15%), constipation (13%) and gastroesophageal reflux disease (GERD) (12%) (Figure 3A). Extra-intestinal manifestations, alone or in combination with gastrointestinal symptoms, were detected in 45% of CD patients (Figure 3B). Frequent findings were anemia (34%), cryptogenic hypertransaminasemia (29%) and recurrent miscarriages (12%). A few CD patients showed IgE-mediated allergy (9%), often characterized by positivity for specific IgE to graminaceae and mites. A small number of subjects showed headache (5%) and fibromyalgia-like symptoms (2.2%). Iron-deficiency with low levels of ferritin was found in 85% of patients with anemia, which was also related to folic acid malabsorption. Bone densitometry, performed in two-thirds of the 770 CD patients, revealed a condition of osteopenia/osteoporosis in 52% of cases, often associated with 25-OH Vitamin D3 low levels.

### Serological tests

A total of 744 (97%) out of the 770 CD patients were positive for IgA anti-TG2, whereas 91% of the same patients were IgA EmA positive. EmA detection was always coincident with anti-TG2 positivity. Of the 26 patients negative for IgA anti-TG2 and EmA, 15 had selective IgA deficiency and all of them were positive for IgG anti-TG2 or DGP. Only 11 CD patients (9 females) were seronegative. The median age at CD diagnosis was significantly higher in the seronegative vs. seropositive patients (49 vs. 36 years, *P* < 0.005). Compared to seropositive cases, CD patients testing negative for serology were all characterized by a significantly higher prevalence of classical phenotype (*P* < 0.001). Four seronegative patients showed a positivity for first generation gliadin antibodies of IgG class (1 case also associated with IgA), nowadays no longer considered serological CD markers (Table 1).

**Table 1:** **Clinical presentation, histology, genetics and associated disorders in patients with seronegative celiac disease**

**Pts**	**Gender**	**Age at diagnosis**	**Clinical phenotype**	**HLA-typing**	**Duodenal biopsy**		**Anti-TG2**	**AGA**	**Associated disorders**
					**Untreated**	**After GFD**	**EmA DGP**		
#1	Male	58	Classical	DQ2+	3c	1	Neg	IgG + ve	Autoimmune thyroiditis
#2	Female	34	Classical	DQ2+	3a	1	Neg	IgG + ve	PBC
#3	Female	37	Classical	DQ2+	3c	1	Neg	Neg	None
#4	Female	75	Classical	DQ2+	3c	1	Neg	Neg	None
#5	Female	45	Classical	DQ2+	3a	1	Neg	Neg	None
#6	Female	55	Classical	DQ2+	3c	1	Neg	IgG + ve	Gluten ataxia
#7	Male	61	Classical	DQ8+	3b	1	Neg	Neg	Peripheral neuropathy
#8	Female	48	Classical	DQ2+	3c	1	Neg	Neg	None
#9	Female	49	Classical	DQ2+	3a	1	Neg	IgG + ve; IgA + ve	Autoimmune gastritis
#10	Female	30	Classical	DQ2+	3b	1	Neg	Neg	None
#11	Female	63	Classical	DQ2+	3c	1	Neg	Neg	None

Note: Anti-TG2: tissue transglutaminase antibodies, EmA: endomysial antibodies, DGP: deamidated gliadin antibodies, AGA: gliadin antibodies, GFD; gluten-free diet, PBC; primary biliary cirrhosis; duodenal biopsy scored according to Marsh-Oberhüber classification: "3a", partial; "3b", subtotal; "3c", total villous atrophy; "1" indicates increased intraepithelial lymphocytes.

### Duodenal histology

Villous atrophy was found in 670 (87%) of 770 CD patients. Total (3c) and subtotal (3b) villous atrophy were observed in 36% and in 26% of cases, respectively, whereas a partial (3a) atrophy was identified in 25% of patients. The remaining 100 patients (13%) had a histological pattern characterized by an increased number of IEL (lesion type 1). These minor lesions were consistent with a potential CD, confirmed by HLA-DQ2 and/or -DQ8 and IgA anti-TG2 and/or EmA positivity. A high number of potential CD were first-degree relatives of CD and type 1 DM patients. Only symptomatic cases of potential CD started GFD, whereas subclinical cases continued to eat gluten. Five out of the 100 potential CD cases showed disappearance of EmA and anti-TG2 on a gluten containing diet.

### Associated disorders

Autoimmune thyroiditis was found in 26.3% of CD patients, of whom about half showed hypothyroidism. Dermatitis herpetiformis was found in 4% of CD cases, whereas type 1 DM was detectable in 3% of patients. Other associated diseases included neurological disorders, i.e. gluten ataxia, cryptogenic epilepsy and peripheral neuropathy (all together 2.2%); IgA deficiency (1.9%); autoimmune liver disorders, such as primary biliary cirrhosis and autoimmune hepatitis (1.8%). Finally, connective tissue disorders, mainly Sjӧgren syndrome and systemic sclerosis were diagnosed in 1.7%. Chromosomal disorders coexisted in 15 CD patients, i.e. 12 Down and 3 Turner syndrome. Only three celiac patients were affected by inflammatory bowel disease (1 case of Crohn’s disease ad 2 of ulcerative colitis).

### Non-responsive CD

A total of 654 (85%) of the 770 CD patients underwent a clinical and biochemical follow-up every 18 months. The follow-up duration ranged from 18 months to 14 years (mean 5 years). The response to GFD was regarded as good in 514 patients (79%), whereas the remaining 140 CD cases showed a poor response to GFD and were labeled as non-responsive CD. The main causes of non-responsive CD were poor compliance with GFD (40%), irritable bowel syndrome (20%), GERD (15%), lactose intolerance (12%), bacterial overgrowth (9%) and complicated CD (4%).

### Complicated CD

Six (0.9%) of the 654 CD patients developed complications during the follow-up (Table 2). Three patients had refractory CD type 1, two had small bowel carcinoma and one developed enteropathy associated T cell lymphoma (EATCL). Patients developing complications had a late diagnosis with a median age of CD at diagnosis significantly higher than that of non complicated CD (53.5 vs. 36 years, *P* < 0.005). The estimated diagnostic delay in complicated CD cases ranged from 5 to 11 years. All complicated CD had a classical phenotype testing positive for HLA-DQ2 (3 cases carried DQ2 in homozygosis). Regarding clinical presentation, classical phenotype was significantly higher in complicated vs. non complicated CD (*P* < 0.001) (Table 2). Of the 6 complicated patients, 5 are still alive and only 1 with small bowel carcinoma died 2 years after surgery and chemotherapy.

**Table 2:** **Clinical and genetic features of the 6 celiac disease patients developing complications**

**Pts**	**Gender**	**Age at diagnosis (yrs)**	**Clinical phenotype**	**HLA**	**Delay in CD diagnosis (yrs)**	**Age at complication (yrs)**	**Complication**	**Outcome**
#1	M	42	Classical	DQ2 + °	8	45	EATCL	Alive, 54 yrs
#2	F	66	Classical	DQ2+	10	70	RCD type 1	Alive, 73 yrs
#3	F	52	Classical	DQ2 + °	5	56	RCD type 1	Alive, 64 yrs
#4	F	48	Classical	DQ2+	6	51	Small bowel carcinoma	Dead, 53 yrs
#5	F	65	Classical	DQ2 + °	11	65	Small bowel carcinoma	Alive, 76 yrs
#6	F	55	Classical	DQ2+	7	59	RCD type 1	Alive, 66 yrs

*EATCL*, enteropathy associated T-cell lymphoma; *RCD*, refractory celiac disease.

°DQ2 homozygosis (DQB1 *0201 on both chromosomes) was established in case #1, #3 and #5.

## Discussion

The present study defines the clinical, serological and histopathological features of CD in a large, single-center series of consecutively diagnosed patients. Our data highlighted a significant upward trend of diagnoses over time. In the last 5 years the number of CD diagnoses was one and half times higher than that in the previous ten years. The clinical presentation of CD changed over time with a marked decrease of the classical phenotype (from 47.2% in the first 10 years to 13.3% in the last five years) and with a striking increase of the non-classical and subclinical phenotype which characterized the onset of CD in more than 86% of cases in the last five years. CD was more frequently found in females with a female/male ratio of 3.5:1, a figure that confirms well established data [19]. The median age of CD at diagnosis was between the third and the fourth decade of life, but 5% of our patients were diagnosed in the elderly. These late-onset cases, which were not explained as diagnostic delay, confirm that the disease can occur at any age [20]. CD patients were clinically stratified on the basis of the Oslo classification and, accordingly, the vast majority (79%) were symptomatic. One third showed the classical form of the disease characterized by diarrhea and malabsorption, while the remaining two thirds were labeled as non-classical complaining of atypical gastrointestinal and extraintestinal manifestations. Notably, diarrhea should be no longer regarded as the cardinal symptom of CD [13]. Although there are no comparative studies on the prevalence of symptoms/manifestations (e.g., bloating, alternating bowel habit, constipation and GERD) in functional bowel disorders and CD patients, we demonstrated such symptoms in about 20% of cases suggesting a link between gastrointestinal functional impairment and CD. Confirmatory screening tests for CD should be proposed to patients with functional bowel symptoms, in particular to those people living in areas with high CD prevalence [21]. Frequent extraintestinal manifestations raising suspicion of CD were anemia (due to iron and, less frequently, folic acid deficiency), cryptogenic hypertransaminasemia and recurrent miscarriages. An unexplained osteopenia/osteoporosis was an indicator of CD, being present in more than 50% of cases. One fifth of the 770 CD patients were classified as having subclinical CD. This subgroup has progressively increased in recent years due to the use of serological screening. Interestingly, the age at CD diagnosis was significantly lower in subclinical than in symptomatic CD, thus suggesting that serological screening detects CD earlier than symptoms.

Our series confirmed that IgA anti-TG2 were more sensitive than IgA EmA for the diagnosis of CD [2]. Other important findings included that IgA EmA positivity was never found in cases without IgA anti-TG2 [22]; about two thirds of the IgA TG2/EmA negative cases had IgA deficiency (and tested positive for IgG anti-TG2/DGP) [17] and, finally, that seronegative CD was identified only in a small subset of patients (1.4%) [23]. Seronegative patients showed peculiar aspects not fully reported in previous studies [24]. First, compared to seropositive CD, seronegative disease was significantly associated with a classical phenotype. Thus, in all malabsorptive patients a diagnosis of CD should be always ruled out by small bowel biopsy regardless an unsupportive serological profile. Secondly, seronegative CD was characterized by a more pronounced prevalence of the female gender (F/M 4.5:1) and a significantly higher median age at diagnosis (49 years) than seropositive CD (F/M 3.5:1 and 36 years). Thirdly, a close association with autoimmune disorders including primary biliary cirrhosis, autoimmune gastritis, Hashimoto's thyroiditis, peripheral neuropathy and gluten ataxia was found in seronegative patients. Finally, a proportion of otherwise seronegative CD patients tested positive for IgG AGA, a notoriously aspecific marker of CD [25], which, however, in the presence of villous atrophy may be clinically useful to address the diagnosis [26].

Compared to previous studies showing frequent flat mucosa [27], our data showed that only 62% of CD patients had severe intestinal damage (type 3b, 3c), whereas partial atrophy (type 3a) was found in an increasing percentage of cases (25%). The latter finding may be the result of early detection of CD in a stage when intestinal damage has not yet peaked. Furthermore, in parallel with partial intestinal atrophy, the number of potential CD cases (13%) was also high. The possible disappearance of serological markers in patients with potential CD on a gluten-containing diet particularly in subclinical patients, suggests a cautionary approach before starting GFD [28]. Another aspect pertaining to histopathology is related to the new guidelines emanated by ESPGHAN. Following these criteria it is now possible to skip histopathologic evaluation and to establish CD diagnosis in symptomatic children and adolescents testing positive for high titer anti-TG2 (more than ten folds above the cut-off) and confirmed by EmA and HLA-DQ2 and/or -DQ8 positivity [29]. Our series indicates that the ESPGHAN criteria, although quite interesting and certainly useful in the pediatric age, cannot be applied in adult CD cases [30,31]. In fact, a minority of cases with high titer anti-TG2 (>10 times the upper normal limit) had non-atrophic mucosa being classifiable as a potential CD, implying that in adult patients a firm diagnosis should always be established by means of small intestinal biopsy.

Our data confirm and expand the concept that CD can be associated with a number of autoimmune diseases [32]. A possible explanation for this spread of autoimmunity occurring in CD may be the ubiquitous distribution of TG2 in many other organs and tissues besides the small bowel [33]. Our study indicated that the most frequent condition associated with CD was Hashimoto's thyroiditis, found in about in one fourth of cases, with half of them developing clinical hypothyroidism. Dermatitis herpetiformis and type 1 DM were detected in 4% and 3% of CD patients, respectively, both percentages in line with previous studies [34,35]. Concerning type 1 DM, previous data have shown that it rarely occurs in CD patients on a strict GFD, suggesting that gluten withdrawal may exert a protective role [36]. Brain and liver autoimmune disorders were closely associated with CD as shown by gluten ataxia, peripheral neuropathy, epilepsy (notably resistant to pharmacologic treatment), primary biliary cirrhosis and autoimmune hepatitis identified in our CD patients [37-39]. Concerning connective tissue disorders our data confirmed that Sjӧgren syndrome and, to a lesser extent, systemic sclerosis were closely related to CD [40,41], whereas, as previously reported, systemic lupus erythematosus and rheumatoid arthritis were rarely detected in CD [42,43]. As previously demonstrated, Down and, to a lower extent, Turner syndrome were the two chromosomal disorders most commonly associated with CD [44,45].

An emerging entity is the non-responsive CD, which includes patients with an unsatisfactory response to GFD [46]. In our study we evaluated the response to GFD in 654 CD patients, followed-up for a period ranging from 18 months to 14 years. About 20% of them were labeled as non-responsive CD due to poor compliance to GFD, functional gastrointestinal disorders (e.g. GERD and irritable bowel syndrome) as well as food intolerance, e.g. to lactose. Another cause of non-responsive CD reported in the literature, i.e. CD misdiagnosis, was not identified in our series since all patients had been diagnosed according to well-established criteria [47]. The lack of response to GFD can be also due to the onset of complications in the natural course of CD. For many years the frequency of complicated CD has been overestimated with data ranging up to 10% of the total number of celiac patients. Recent data, however, clearly demonstrated that complications occur in about 1% of CD patients [18,48] and, in the present study, only 0.9% of CD patients developed a complication including 3 cases of refractory CD, 2 small bowel carcinoma and 1 case of EATCL. The risk for the development of complications was higher in patients with a late recognition of CD and with a significant diagnostic delay. Complicated CD showed a significantly higher median age at diagnosis than non-complicated CD. Moreover, the occurrence of complications in CD patients was significantly related with an onset of CD characterized by the classical form with diarrhea and malabsorption, suggesting that non-classical and subclinical forms are at a lower risk of developing complications. CD patients with HLA-DQ2 homozygosis display a higher risk of developing complications [49]. Surprisingly, the prognosis of complicated CD in our series was better than expected with 5 of the 6 patients still alive after a mean follow-up of 10 years.

Possible limitations of this study concerned its retrospective nature which might have biased the data analysis as well as the lack of information on pediatric cases of CD.

## Conclusions

In conclusion, this study detailed the clinical features, serology and histopathological data in a very large series of adult CD patients diagnosed in a single referral center over a 15 year time frame. The results of this series corroborated and expanded previous evidence regarding: *i*) the clinical presentation of CD according to recently defined criteria (i.e. non-classical and subclinical prevailed over the classical forms); *ii*) the possibility of establishing an early diagnosis (i.e. when the disease is still in a preclinical/paucisymptomatic stage), hence justifying an increased number of potential CD cases; *iii*) the different serological and histological patterns of the disease, which can help in the diagnostic work-up; and *iv*) the identification of autoimmune disorders commonly associated with CD. Finally, the follow-up of our CD patients showed that a high proportion of cases (about 20%) could be labeled as non-responsive CD, mainly because of low compliance to GFD or concomitant functional gastrointestinal disorders. The lack of responsiveness can be related to complications, which, although rare, require an early diagnosis in order to avoid a poor outcome.
